# Seeking information and services associated with reproductive health among rural Peruvian young adults: exploratory qualitative research from Amazonas, Peru

**DOI:** 10.1186/s12978-024-01769-2

**Published:** 2024-03-13

**Authors:** Molly F. McGuire, Emma Ortega, Radha Patel, Valerie A. Paz-Soldán, Amy R. Riley-Powell

**Affiliations:** 1 Department of Global Health Systems & Development, School of Public Health and Tropical Medicine, Tulane University, New Orleans, LA USA; 2 Department of Tropical Medicine and Infectious Disease, School of Public Health and Tropical Medicine, Tulane University, New Orleans, LA USA; 3 Department of Medicine, University of Tennessee, Knoxville, TN USA; 4 Institute of Development Studies, University of Sussex, Brighton, BN1 9RE UK

**Keywords:** Information seeking, Sexual & reproductive health, Health information, Adolescent health, Rural

## Abstract

**Background:**

Sexual and reproductive health (SRH) literacy allows young adults to make informed decisions about health outcomes. In Peru, roughly one fifth of the population lives in rural areas, and little is known about where young adults in rural areas get their SRH information. The aim of this study was to identify what motivates and influences young adults to seek information and care related to SRH in three rural communities in the highlands of Northern Peru.

**Methods:**

Five gender-stratified focus group discussions with a total of 24 participants, and nine follow-up interviews were conducted to generate in-depth narrative data and triangulate data from the target group. Participants were women and men aged 18–24. The focus group discussions and interviews explored sources of reproductive health information, the role of informal social networks, barriers to care, and primary health concerns of the target population.

**Results:**

Main findings include: (1) The two greatest perceived SRH risks were unwanted pregnancy and abnormal discharge; (2) There appears to be limited concern about HIV or other sexually transmitted infections in the narratives; (3) There is a low quality of information concerning SRH, with discrepancies between the genders; (4) A broad spectrum of sources for SRH information were cited, including Internet, traditional healers, and specialized care; and varied by gender and life experience; (5) Having trust in the information source was the primary variable associated with uptake of services and/or access to information for both men and women. However, men reported more embarrassment around seeking services and information, whereas women faced more physical barriers.

**Conclusions:**

There is a lack of SRH information among young adults in some communities in the northern highlands of Peru. Both schools and health centers were noted as being trusted and established information sources for all genders so could be a key resource to explore as a way to disseminate information.

## Plain Language Summary

Access to reliable and accurate information about sexual and reproductive health (SRH) allows people to make educated and informed decisions about their reproductive choices. This can lead to more desirable outcomes, including lower rates of teen pregnancy. Access to SRH information is not universal and can be impacted by various factors including politics, religion, and culture. Much of what is known about SRH in the rural regions of Peru comes from the national census data, which is collected through surveys and does not allow for open ended answers. Our study held in-depth interviews with young adults in the Amazonas department of Peru to get a better understanding about what SRH concerns they have and where they learn about SRH information. The study findings showed a limited level of SRH knowledge in this community in Peru. Having trust in a person is the most important factor participants considered when deciding where to seek information from. Participants cited parents, particularly mothers, teachers, and the internet as trusted SRH information sources, none of which were necessarily reliable. There was widespread agreement among the participants, that their opposite gender had better access to SRH information. Though results varied between male and female participants, with men being less willing to seek information and women facing more physical barriers to access. In conclusion, more reliable and consistent options for adolescents to learn about SRH are needed. Established networks of trusted sources, like schools and clinics, could be utilized when considering dissemination.

## Resumen

**Fondo:**

Conocimiento sobre la salud sexual y reproductiva (SSR) permite a adultos jóvenes tomar decisiones informadas sobre su salud. En el Perú, aproximadamente un quinto de la población vive en áreas rurales y se sabe poco acerca de dónde adultos jóvenes en estas zonas obtienen su información sobre SSR. El objetivo de este estudio fue identificar qué motiva e influye a adultos jóvenes a buscar información y cuidados relacionados a su SSR en tres comunidades rurales de la sierra norte de Perú.

**Métodos:**

Se realizaron cinco grupos focales con un total de 24 participantes, estratificados por género y nueve entrevistas para generar narrativas detalladas y triangular información del grupo objetivo: mujeres y hombres de 18 a 24 años. En los grupos focales y entrevistas se exploró sus fuentes de información sobre SSR, su uso de redes sociales informales, sus barreras para recibir atención de SSR y sus preocupaciones relacionadas a salud primaria.

**Resultados:**

Las principales conclusiones fueron: (1) Los dos mayores riesgos de SSR percibidos fueron el embarazo no deseado y el flujo anormal; (2) No se observó preocupación por contagiarse con el VIH u otras infecciones de transmisión sexual; (3) Hay una baja calidad de información sobre SSR, con discrepancias entre géneros; (4) Se usan una variedad de fuentes de información sobre SSR, incluyendo Internet, curanderos tradicionales y atención especializada; y esta varía por género y experiencia de vida; (5) Tener confianza en la fuente de información fue la principal variable asociada al uso de servicios y/o acceso a la información para hombres y mujeres. Sin embargo, los hombres se avergonzaban más de buscar servicios e información, mientras que las mujeres enfrentaban más barreras físicas.

**Conclusiones:**

Hay una falta de información sobre SSR entre adultos jóvenes en ciertas comunidades de la sierra norte del Perú. Las escuelas y los centros de salud fueron identificados como fuentes de información confiables y establecidas para todos los géneros, por lo que podrían ser un recurso importante para explorar como una forma de difundir información.

## Background

Young adults aged 15–29 comprise 25.2% of the population of Peru and make up a significant part of the work force [1]. Nearly 19% of the young adult population live in rural areas, where knowledge about fertility, family planning, and routine medical exams is lower compared to their urban counterparts in Peru [1]. The majority of the research on sexual and reproductive health (SRH) among young adults in Peru has been conducted in urban areas and our knowledge of behaviors and attitudes towards sexual and reproductive health in rural areas is less well understood [2–7]. A lack of contextually and culturally relevant public health messaging has been found to influence health-seeking behaviours in the Peruvian context [8]. Improving our understanding of how young people seek information on SRH has the potential to help ensure adolescents in rural areas can access the same knowledge as their urban counterparts, through contextually relevant information.

Amazonas is a rural mountainous region in northern Peru. Most families in this region are subsistence farmers with approximately 40% of people aged 15 years and older having completed secondary education [9]. In 2018, 21.2% of adolescents 15–19 years old in Amazonas were or had been pregnant, which is higher than the national rate of 12.6% [1].

In the 2016 national census 86.9% of women ages 15–49 living in Amazonas report not having spoken with a health professional about family planning in the last year [10]. However, 68.3% report having sex in the last 4 weeks, which is higher than the national rate of 57% [10]. Reports of intimate partner violence in Amazonas are in line with the high rates seen throughout Peru (and Latin America) with 61.6% of once married women reporting some form of subjugation by their partner, and 25.7% reporting physical harm by their partner [10–12]. This is important to consider as prevention and response to violence are closely linked with SRH services.

With regards to seeking information, support or treatment, there is evidence to suggest informal networks and traditional healers both play a significant role in this region. Informal networks, such as friends and family, play a role in the flow of sexual and reproductive health information to and from young adults in rural Peru. For example we know that family obligations are an important factor for women when deciding to seek breast care in urban Peru [13], and that young adults may rely primarily on their parents for sexual health information, especially their mothers [14]. For treatment, available data suggests that young people in Amazonas relies both on modern medicine and local traditional beliefs about health and healing, with 18.4% of women surveyed reporting that they had sought treatment or advice about sexually transmitted diseases from traditional healers [10].

Including informal networks, Winert et al. established four categories for sources of health information, which will guide this analysis.

*“Health-seeking represents the range of activities that individuals undertake to promote or restore health, including health promotion/protective activities, ****self-management of health problems****, ****use of informal resources such as the family network, and use of formal professional resources.” (Weinert, 1994)*** [15]

Formal professional networks are often cited as the “go-to” resource in hypothetical healthcare situations and are the most widely utilized sources at the national level [16]. However, there is often a discord between knowledge and confidence to use formal professional networks. For example, in Amazonas, 89.7% of women know health centers provide condoms, however only 64% of women surveyed feel confident they could acquire a condom [10]. Societal attitudes toward sexual and reproductive health can also influence the decision to seek health information in Peru. Information about SRH is not consistently offered in schools, with one study finding that only 9% of students received comprehensive sexual education in school addressing all 18 identified topics. In fact, less than half the students reported receiving a lesson on “contraception and unwanted pregnancy” [14].

Existing literature on adolescent SRH in Peru can be classified as either quantitative data, largely census survey statistics, or studies focusing solely on urban areas. To our knowledge, there are no existing quantitative studies focused on understanding SRH opinions and practices in the rural area of Amazonas, Peru, creating a gap in an area that is important to study as a fifth of the adolescent population of Peru lives in a rural area. In this project we sought to address this gap by employing an exploratory approach to understand more about adolescent experiences with SRH in Amazonas, a rural area of Peru.

## Methods

### Study setting

Data collection took place in three rural communities in the Northern state of Amazonas in Peru: Tingo, Magdalena, and Choctamal. All three towns have public health facilities, local government representation, and three or more health-related social programs available to its citizens. Choctamal is furthest from the state capital and city of Chachapoyas compared to Tingo and Magdalena (52.2 km versus 39 and 38.6 km, respectively). Tingo has a larger health center with more exhaustive health services; because of this, all medical referrals from these communities must go through the Tingo health facility to advance the case. This research site was chosen because of the limited information available about young adult sexual health knowledge and practices in the area.

### Study team

The study team all speak “Peruvian Spanish” and have familial ties and/or have spent significant time living and working in Peru. The first author has pre-established relationships and ties to the study setting from several years of community engagement and living in the region. The research question emerged as part of an ongoing project and the research design was developed with the experiences and comfort of the participant group in mind. The first author was supported by Dr. Paz-Soldán, a Peruvian-American social scientist with ~ 20 years’ experience conducting research in various regions of Peru and mentoring early career researchers. Throughout the data collection, analysis and write-up process, the study team engaged in reflective practice as part of the analytical process.

### Participant recruitment

Purposive sampling techniques [17] were used to recruit young adults (18–24) living in Tingo, Choctamal, and Magdalena. Participants were approached in their homes by the researcher and invited to participate, and in some cases, those recruits assisted in the recruitment of other eligible community members by telling them about the study and inviting them to participate.

### Data collection and analysis

From August 2014 to October 2014,^1^ three female (n = 14) and two male focus group discussions (n = 10) were conducted. Focus group discussions (FDGs) were conducted in neutral environments, such as the local high schools, offices in the community centers, or health posts [18]. Each FGD session was attended by a note-taker, was audio-recorded, and lasted no longer than two hours. All focus groups followed the same interview guide, developed by the researcher. The interview guide covered topics including the main health concerns for the community and specifically for peers their age, knowledge and sources of sexual and reproductive health information, and barriers to healthcare.

Nine personal private interviews (PPIs) were also conducted directly following the focus groups with five female and four male participants from the FGDs who were willing to participate. The results of which corroborate the FGD data and provide further insight into the research question. All study procedures occurred in Spanish, and study personnel were local professionals hired and trained by the researcher. The study tools were pre-tested in a teaching facility in Chachapoyas, which was not included in the study.

After each FGD, the researcher met with personnel to generate a report. The audio-tapes of both FGDs and PPIs were transcribed and coded by the researcher, and corroborated by the report. The transcripts were coded inductively and deductively. Winert’s four categories for sources of health information were primarily used to guide the data coding and new codes were added to reflect themes which arose during coding.

### Ethics

The Tulane University Institutional Review Board approved this research for ethical content and management (IRB# 553385). Per the IRB recommendation, the research proposal was reviewed and discussed with local representatives from the Ministry of Health and the Ministry of Education. Letters of approval were obtained from the local Ministry of Health and Ministry of Education offices in Amazonas prior to starting the study. According to Peruvian law, mandatory reporting of rape or sexual trauma of minors in Peru is required from health and school staff. Although 18 was the minimum age for study participation, we implemented contingencies with the facilitators to remind participants throughout the discussions not to discuss real cases involving minors. We included the telephone number of the local “Ministry of Women and Vulnerable Populations” who is responsible for rape/violence cases to the consent form that was read to participants and repeated at the end of each focus group. The case may be taken up with local authorities (Public Defense Attorney, health professionals, organizations for victims of abuse, etc.) if the victim is willing and wants to disclose his or her case. We also emphasized that therapeutic counselling was available to participants via the national health system.

All participants gave informed consent to participate prior to the gender-stratified focus groups.

## Results

The study sample included 24 young adults, aged 18–24, where the average age of female participants was 23, and males was 20. All female participants had at least one child and none of the male participants reported having children (Table 1). Results can be categorized into four main topics: primary health concerns, sexual and reproductive health knowledge, sources of information and services, and self-management of sexual and reproductive health concerns.

**Table 1:** Population characteristics

Characteristic	Male	Female
Total participants	10	14
Mean age (range)	20 (18–23)	23 (20–24)
Relationship status n (%)		
Single	9 (90)	5 (36)
Married	0 (0)	2 (14)
Cohabitating	0 (0)	7 (50)
Other	1 (10)	0 (0)
Children n (%)		
0	10 (100)	0 (0)
1	0 (0)	9 (64)
2	0 (0)	5 (36)

### Primary health concerns

#### Community-wide primary health concerns

Health concerns which participants mentioned without prompting included general body ache, digestive problems, developmental or growth issues (height, weight), infectious diseases, and acute symptoms, such as coughs or fever (Table 2). In the female FGDs, women mentioned headaches and stress among their top general health concerns for people aged 18–24. Women elaborated on their limited social and physical mobility within their communities and familial obligation as a source of chronic stress.

*“[There is stress] because you have to know everything about taking care of the house: sweeping, washing, cooking and sometimes it’s all for nothing and it gets thrown out anyway” (F2p7D)*

**Table 2:** Primary non-SRH concerns of informants by gender and data collection method

	Community-wide primary health concerns
Female FGD	Migraines/headaches (4), Stress (3), Typhoid/fever (2), gastritis (2), urinary infections (2), acne, depression, flu/head colds, Leishmaniasis, back pain, haemorrhages, hernias, stunting, varicose veins
Female PPI	Hygiene, unemployment
Male FGD	Accidents (2), alcohol (2), addiction, cough, fever, gastritis, Leishmaniasis
Male PPI	Cancer, diabetes, fever, flu, Leishmaniasis, pollution, problems with parents

(): number of times mentioned when asked about health concerns

In the male FGDs accidents and alcoholism were among the top general health concerns. They also spoke at length of structural concerns related to health including access, poor quality of service, the lengthy referral system, and a lack of common services such as ambulances.

*Sexual and Reproductive Health Concerns.* The SRH-specific concerns of male respondents centred around unwanted teenage pregnancy (Table 3). They reported a shared fear that a partner might lie to them about becoming pregnant and articulated the consequences of teenage pregnancy. In response to the PPI topic about primary SRH concerns, one interviewee offered:

*“Teen pregnancy [is the greatest health concern for people my age]. They give health talks but kids don’t pay attention and end up pregnant.” (ME3)*

**Table 3:** Primary sexual and reproductive health-related concerns solicited of informants by gender and data collection method

	Sexual & reproductive health concerns
Female FGD	Abnormal discharge* (2), Cervical cancer (2), no weight gain during pregnancy
Female PPI	Unwanted pregnancy (2), abortion, suicide
Male FGD	Unwanted teenage pregnancy (2)
Male PPI	Abnormal discharge* (2), unwanted teenage pregnancy (2), teenage pregnancy

*Discharge, ejaculation or *granos*, refers to STI as this is how they are taught to identify STIs

In the female FGDs, SRH-related concerns mentioned by women included cancers and sexually transmitted infections (STIs). They concluded that these were most likely spread through cheating partners.

*“The discharge is probably because of the numerous sexual relationships they have...usually between 18-24, well, because at that age most people are living together but sometimes they break up and get with another girl. That’s how they get infected with illness.” (F3D)*

“Unwanted pregnancy” by name was only mentioned once among female groups. Yet, the implications of unwanted pregnancy were frequently discussed:

*“They don’t let you study...if you are working towards a career, they won’t let you. You leave everything for your kids. Like, it’s a step backwards. One, for example, wants to study or go to work, but it’s not the same anymore. And if you do go to work, everything is for your kid. Like, when you want to do something with your money, you can’t. Everything goes to buy his clothes, food, milk, etc. Until all the money is gone and you can’t buy anymore.” (F1p4C)*

*“It’s the worst thing that can happen [when you get pregnant by someone else]. And sometimes that person doesn’t take responsibility. They forget about you...sometimes that can happen with women. We women are so weak, more so than men. Men are stronger.” (F1p5c)*

Participants only felt comfortable discussing certain health concerns for people their age in private. The PPIs were the only time males discussed STIs and females discussed unwanted pregnancy.

### Sexual and reproductive health knowledge

Respondents were asked what sexual and reproductive health most likely refers to. Most women agreed that sexual and reproductive health refers to pregnancy and childbirth, *“to plan our lives and how many children to have”* (FGD F1), while men mostly understood it to relate to sexuality, sexual illness, and conduct: *“it means sexual relationships with your female partner”* (M2p4A). HIV/AIDS only came up twice during the study. *“There is no AIDS here”,* said one male interviewee. STI comprehension was limited, and not referred to by name but rather by descriptive symptoms, such as “*granos*” (bumps) or discharge (Table 3).

*“For example, in my case, I know what STIs are but I don’t know what disease they cause. I read and it says “STI”, but I don’t really know what that means. I know it’s a disease but what could it be?” (F1p12B)*

The respondents who had lived outside of their communities expressed greater fluency in SRH topics and more confidence in their contribution to the discussion.

### Sources of information and services

Sources of SRH services or information varied by gender, reproductive status, and depth of experience outside of the community. The majority agreed one could seek help or information from people they had *confianza* with, or trusted. Passive exposure to information was mentioned, such as radio, handouts, flyers, billboards, etc., but will not be addressed in this analysis.

#### Informal networks as a source of information and services

In hypothetical situations, people in the community would refer to their informal networks as a first line of information: *“it’s something personal that sometimes you can tell someone you trust*” (F1p9E)**.** Informal networks typically consisted of family, friends, and respected community members. Parents, more often mothers, were generally seen as a trustworthy resource for both male and female participants, *“My mom taught me how to place the pad in my underwear. I learned from my mom” (F3A).*

In some cases, women also rely on friends,

*“In my case, my mom isn’t around anymore. I have my best friend who I tell everything. If I am upset or I want to cry, I go to her. When I go to her she always listens. Sometimes she also has problems and she tells me everything, too. But sometimes she’s not home and I have to wait until she gets back.” (F1p10)*

However, we also found that friends grow apart, and social support networks erode,

*“When you get engaged, your girlfriends start to distance themselves from you. To find a girlfriend these days is very difficult.” (F1p5,10B)*

*“Sometimes we don’t get to talk [with other women]. We just don’t know anything about them.” (F2p8C)*

Women with children tended to rely most on their partners as their source of information or services for SRH related issues.

*“...sometimes when you have a partner, um, you eventually gain trust as a couple more than you had with your family. When I lived with the father of [my daughter] I trusted him with everything. Even things my mom didn’t know or my own family- he knew. You don’t feel ashamed anymore to tell him, isn’t that true?” (F1p10)*

Men reported confiding in friends and teachers more than any other source of information or services, though this was contingent on their individual self-esteem.

*“If you have low self-esteem, you keep [your health problem] secret. If you have high self-esteem, you can seek help from your friends.” (M1p3E)*

If they could not confide in friends, men cited teachers and educational courses as their sources of knowledge. The trusted relationship between teacher and student appeared to be mutual:

*“Aside from being my professors, they are my friends. If I disrespect him, he won’t trust me anymore...” (ME5)*

#### Perceived differences in access to SRH information between genders

When asked about gendered differences regarding access to SRH information or services, women acknowledged self-imposed barriers to access SRH information and services*, “we don’t inform ourselves well sometimes” (F1p11B*). They generally agreed that men know more about SRH and have easier access to information and services through community friendships.

*“Sometimes [men] make friends [with the health personnel] in our neighborhoods. They can ask them, if they are male, because sometimes they play sports together. They meet at the soccer field, make friends, and talk. They are less ashamed.” (F1p20B)*

However, male responses presented a dichotomy, with the belief that women know more about SRH.

*“Yes, I’ve heard of family planning, but more than that, no. It’s a woman’s thing” (ME5)*

Part of this belief stems from the idea that women have more clinic visits due to pre- and post-natal visits for their children, so women have a better relationship with the health providers:

*“Men have no pretext to go to the health center like women do”* (Male PPI).

Several of the female participants discussed how relationship dynamics affected their access to SRH information and services. Many women in this area, especially early in their marriages,^2^ feel constrained by heteronormative gender roles. In this context for women to fulfil a traditional female role they would be subject to limited social/geographical mobility, dedication to household duties, and an expectation to be a wife and a mother. Simultaneously, young women can experience a sense of isolation when they move in with a partner, shifting away from their family and other social connections. This new way of life can impact women’s decisions to seek health-related information or services. In several cases women feared their partner would become jealous if they made friends or confided in others about their health issues.

*“Women have to prevent against everything and it’s very difficult [to find information about SRH] because men don’t consider it important and could even be with another woman...” (FE1)*

Women agreed that a common way to secure one’s future and put to jealousy to rest was to become pregnant:

*“Well, when you are single, know that I’m thinking about it, all the boys flirt with you because you’re single. You have no one- no boyfriend. But when you are with someone, they start saying...things. If your husband is jealous, that’s when you can’t afford to have friends. That happens everywhere, not just here. You fight, you take it. Things like this always happen in small towns... The men also, because they are jealous, they want to start a family quickly. They don’t think about it first: why would they? He would say, ‘well, maybe this idiot will leave me.’ Men always fear that. They want to forcefully guarantee the relationship. If you go anywhere, the man becomes jealous: why do you wear makeup? Why do you dress that way? Questions like that, one after another before it turns into a fight. Like, that’s the limit...There are many cases, for example, that the man prohibits you from going to a town party, he says, ‘no, you’re not going’ but you still want to go. But you can’t go, even if you were dead! There are women who don’t protect themselves and become pregnant very quickly. That’s where the abuse begins.” (F1p5A)*

Men did not directly comment on jealousy, but rather said their greatest fear is their female partner would lie to them about being pregnant or cheat on them, feeding into harmful heteronormative stereotypes.

#### Formal professional networks as a source of information and services

Generally, respondents were content with the quality of care and information provided by health center personnel who seemed to know and understand the value of trust and its role in providing successful healthcare.

*“In each institution there is someone who inspires more trust...like, maybe not the doctor or the midwife, but there is someone you can talk to, that you trust. There is always someone you can trust in these institutions, everywhere.” (F1p12C)*

But one female respondent lamented the high rotation of personnel through the system, saying just when you get to know them, a new one replaces them, and you have to build trust all over again. On the other hand, women expressed that the potential shame associated with being seen at the local health facility poses the largest obstacle to seeking formal healthcare services.

*“Sometimes when you are pregnant you are also ashamed. You know why? When you get to the health center, the entire population is sitting there....one feels ashamed just by being there.” (F1p6A)*

*“Whoever goes to the health center to take care of themselves, the day after next people start to talk all over town. This happens in every town.” (F1p6E)*

Several male and female respondents reported negative past experiences with the health personnel in their community. The health personnel made them feel ashamed, unimportant, or uncomfortable.

*“They don’t explain things well...that’s why it’s so shameful. It’s such a headache to go [to the health center] ...sometimes they humiliate you...but at least they attend to you and then you can leave. It’s not a question of sitting with them to chat.” (F1p11,18E)*

*“Some [patients] stay quiet and don’t talk...but if the health post doesn’t offer you their trust, you simply don’t go because sometimes you go and they tell you ‘don’t wash with that’ or the other...but the whole time you’re worried about something else and so you forget to ask your questions.” (F1p18,12b)*

Women who spent time living outside their towns recommended seeking formal healthcare in the city, especially if it was a sensitive health issue.

*“The bad part is, in the small towns, they watch everything that happens. It’s not like the city...in the city no one knows you. So it’s normal [to go there] when you don’t want anyone to know.” (F1p6,7C)*

*“Mostly for me I go to the gynecologist. There I tell him everything that’s going on. Sometimes with their experience they give you advice- they tell you everything- but advice more than anything. Because trusting in family or a friend...can go badly, that’s why. Sometimes the person you know best, you fear most how they will see you with these issues.” (F1p10,17A)*

However, not everyone has this option due to financial constraints and household responsibilities.

*“[If there are symptoms of STIs] you go to the hospital to consult a doctor for a 100% cure. But if you have no money, you go to the health center.” (ME5)*

### Self-management of SRH

Due to the high level of discomfort and embarrassing nature of SRH information and services seeking, self-management emerged as a technique for actively seeking this type of information or dealing with SRH problems.

*“I take care of myself because they can make fun of you [at the health post]. I learned from my own suffering.” (FE2)*

The most popular informational tools for self-management included the Internet and books. Going directly to urban pharmacies were recommended, especially if someone is timid or does not get along with their local health personnel. Participants brought up self-purchasing SRH-related products like condoms, pregnancy tests, baby formula, birth control pills, and lab tests. Bypassing health centers and purchasing the products directly is only an option for those with financial means. Avoiding the health center also means there is a risk that people don't know these products exist or how to safely use them.

*”In the case of pregnancy, there are some people who say, ‘no problem [to do it] without protection. You can just go to the pharmacy to buy the pill that’s for the next day.’ But that’s in the case that you are informed...” (F1p14B)*

*“If they are shy [and thought they might be pregnant], I would tell them to buy a pregnancy test, and yeah, maybe that way you would know.” (F2p11A)*

A few respondents suggested self-managing SRH concerns using natural methods, such as prenatal exercises, in the case of one woman who had spent time in Lima, or use of local plant roots/extracts.

*“For example, for sexual impotence of men, there is a plant up on the mountain. It’s shaped like a penis, the exact same... You put it in water and you drink it. It’s for impotence. They call it the “panty-breaker”. (F1p19B)*

*“Most people use herbs to clean up their abnormal discharge. There are plants well-known for their curative properties.” (FE1)*

During the male PPIs, respondents reported they would be unlikely to seek care or information at all, particularly among young men who are ashamed to utilize health facilities. In one male respondents’ estimate, *“only about 5–10% of guys look for information*” (ME7). Male distancing is a concept that arose as they suggested men would be more likely distance themselves from the SRH concern by keeping it a secret, terminating a relationship, or resorting to alcohol or suicide, as one respondent suggested, *“they could end their life” (ME4).*

*“For young men who don’t talk much [it’s hard to find information about SRH]. They prefer to have the disease rather than telling someone and the shame associate with that. In the case of symptoms of STIs, he wouldn’t say anything. He would become frustrated by his thoughts and could harm himself. It could go badly at work or with his family. He would turn to alcohol.” (ME3)*

Reference books and the Internet were also mentioned as a means of self-managing SRH concerns among both men and women. None of the communities had libraries or Internet available (either publicly or for cost). One female PPI who had lived in Lima suggested *“the Internet could improve access to information about SRH in Tingo”* (FE1). However, other respondents expressed doubts about the efficacy and trustworthiness of the Internet as a resource for SRH information or services.

*“Sometimes [it’s difficult to find information about SRH]. For example, sometimes you can have [access to] Internet, but other times no. And when you know [how to use the Internet and computers] you go to the Internet cabinas- when you know. But when you don’t know you just go to the health center, to the midwife, to ask. (F1p12B)*

*“The Internet is a barrier [to accessing information about SRH]. It could give me right and wrong information.” (M1p3A)*

## Discussion

SRH is an umbrella term and encompasses a broad range of concepts. In this study we have focused on exploring SRH seeking behaviour for young adults 18–24 living in rural communities, a difficult group to reach in rural Peru. To the best of our knowledge, this is the first qualitative study exploring SRH in this rural community, and our results identified several notable themes including health priorities, reliability of resources, vulnerabilities of health center users, limited mobility of women and partner reliance, perceived gendered differences in access to information, and male distancing.

### Health priorities

A taboo on sexual health conditions was evident, such that STIs were a major concern but were referred to using descriptions, like “*granos*” (bumps) and “discharge” instead of attributing a name to them. Not knowing what STIs are or how they manifest poses a public health concern among young people in this area where the population is growing quickly. Bayer frames this lack of knowledge on STIs as an access issue in urban areas such as Lima, specifically that youth lack access to supportive people and structures key to positive development [3]. In Amazonas, 50.9% of women surveyed reported not knowing about STIs [10].

Unwanted teenage pregnancy, which is common in this area, was addressed in various ways throughout the FGDs and PPIs. On the whole, men were able to define, explain prevention and hypothetically react to a teenage pregnancy event. Women, however, never addressed it by name. They fully explored the effects of unwanted teenage pregnancy, such as abortion, financial burden, abandonment of education etc. but never attributed them to the pregnancy itself. This is consistent with national data showing 31.5% of women ages 12–24 in rural areas drop out of school due to pregnancy and marriage [21]. While more information and access to family planning information is needed, this alone will not combat the present structural issues that impact adolescent fertility. Stigma and gender inequality still exist, evidenced by the lack of the word “pregnancy” by females, and is a larger societal issue. Further research outside of the scope of this paper is needed to address this.

### Reliability of resources

Study informants revealed most SRH information comes from resources perceived as trustworthy but often with limited accurate knowledge. Some informants even admitted their main resources, including parents, other family members, and friends, are probably not reliable or accurate. While it is likely this generation of adolescents have received some degree of sexual health education, it is unlikely their parents received accurate sexual health education and are up to date on current SRH information. Older, trustworthy citizens were seen as a resource, which is perhaps a clue about how misinformation is spread throughout the community, once again highlighting the need for more health education centred on SRH.

Although some studies suggest few rural Peruvians rely on the Internet for health information [16], internet use was mentioned as a semi-reliable source of SRH information in almost every focus group. However, access to the internet can be an issue since, as of 2019, only 24% of homes in Amazonas had access to internet [9]. Another common source of internet in rural areas includes Internet *cabinas* (internet cafes) but the closest Internet *cabinas* are in the regional capital of Chachapoyas [22]. Money and ability to travel are a barrier for internet access and highlight the rural–urban digital divide. Though, 85.4% of households in Amazonas have access to mobile phones which could be a possible route to disseminate information [9]. One study found that use of an SMS service to access SRH information brought about greater understanding of contraceptives in Lima youth [23].

Several studies argue that providing differentiated services, both SRH and other, can help alleviate the propagation of misinformation [24, 25]. With emerging health issues, such as Zika virus, which has an impact on reproductive health outcomes, accurate health information is increasingly important and being able to trust health workers is a key part of providing a comprehensive and appropriate health services.

### Vulnerabilities of health center users

It was evident in this study that trusting the source plays a key role in the decision to seek care and information. Although several informants in our study discussed their distrust of the formal healthcare workers, many proclaimed to have strong interpersonal communication with individual workers they deemed trustworthy. A notable barrier to SRH information and services is the stigma women feel when visiting a health center. It has been observed that uncertainty and insecurity impede health seeking among women in Peru for health screens, such as mammography [13]. Many rural women prefer to give birth at home and with a trusted friend rather than a medical professional (compared to urban areas). Medical professional attention during birth is far less common in rural areas (64%) compared to urban areas of Peru (95%). Indeed, 15% of rural births in 2010 were delivered by a friend, and 16% by a midwife (external to the healthcare system) [10]. A review on stigma in healthcare facilities found that some approaches for reducing this stigma include educating healthcare workers about the consequences of stigma, increasing contact between healthcare workers and patients to promote empathy, and employ participatory learning activities between both sides [26]. Based on these findings, we noted a gap for future research. As there are two sides to every story, the perspective of local providers should also be explored. A study in Nigeria looking at adolescent SRH attitudes and practices through the lens of both adolescents and community health providers, found that providers gave specific insights on adolescent health-seeking behavior and local SRH trends [27]. Further research to involve and characterize the experiences of providers would be beneficial in this understudied region, including both clinically trained professionals and traditional healers such as curanderos and parteras.

### Limited mobility of women and partner reliance

Both male and female informants contributed to the shared idea that there are limited health options for women in their communities. Marriages are traditionally patrilocal, which could be a significant distance from the woman’s familial ties and sources of SRH information. Informants discussed a variety of factors unique to young women, including the male partner’s constant need to secure the relationship, the sudden loss of a social network and support, and the social desirability to impress a new husband and in-laws. The physical distance and shift in social support are also why friendships erode. These common life events in rural Peru result in limited mobility for the woman and complete reliance on the new male counterpart. In addition, women in this age group (15–19) have less decision-making power in the home, which has implications for family planning and initiative taking for health benefits, like which type of contraception to use. Injection is by far the most popular contraceptive measure in Amazonas (17.4%) right after abstinence (26.7%) and no method (22%). In 2010, 7.7% of women aged 15–19 reported they do not have the last word in any household decisions, compared to 1.2% across all age groups [10]. Future programming in SRH in rural Peru should take the isolation of new mothers into account when considering dissemination of information.

### Perceived differences in access to information

Women in this study assumed that men knew about health issues and have better access to health information. They suggested men might seek SRH information through friendships made at public events such as soccer matches, which women do not participate in. Men themselves, however, reported rarely relying on friends for SRH information. In fact, men felt women have better access to SRH information. They suggested through the increased exposure to formal healthcare settings during pre-natal and wellness visits, women could develop a closer relationship with the health center staff to procure information. This is consistent with survey data from a Peruvian study that asked who would be more likely to seek help for chest pain symptoms. Female survey respondents were much more likely to say a man would seek help, and women are less likely to seek help for those symptoms [28]. Although, as mentioned earlier, only a small percentage of women in Amazonas (19.3%) spoke to a provider about family planning [10]. Based on census data, women in Amazonas are more likely to reach out to someone close to them (43.1%) than a seek help from an institution (26.6%) [10]. Because of this perceived relationship women have with health center staff, it is likely other groups feel marginalized and that services do not extend to them, such as adolescents or men. The belief that young men and women believe others know more about SRH highlights the need for more education and resources for this age group.

### Male distancing

Reliance on self-managed care for men and distancing habits leads to self-limiting health options for men in terms of health information and services. Multiple young men in our study suggested their teachers played a role in their development during adolescence and were largely relied upon as a trustworthy source of information. This is consistent with global studies in health seeking behaviour [29, 30]. Making men aware of the benefits of modern contraception, would more likely lead to adoption of healthy SRH as men are the key decision-making partners in many cases. Women in the study even questioned, *“why do they only explain these things to the women and not to the men?”* (F1p23B).

These results suggest that maximizing collaboration through school-based initiatives, even something as simple as providing professors with relevant materials, may lead to increase self-efficacy for men and better health outcomes in the target population [2, 4, 6]. Although the Peruvian Ministry of Education (MoE) has made strides in incorporating health education into the curriculum, it still faces significant barriers in this region. Many are abstinence-only programs, or irrelevant, over-simplified, or incomplete, as reported by both men and women in this research. When asked, the majority of teachers interviewed requested additional training and materials for teaching SRH in schools [14]. In 2017, the MoE modified the educational curriculum with changes to promote gender equality, acceptance for all sexualities, and improved sexual education. In response, the social movement “Con Mis Hijos No Te Metas” was created to oppose these changes and instead prevents progressive views on gender norms and SRH from being promoted [31]. High teacher rotation also results in less consistent information, and fewer opportunities for trust building, which is critical for integral sexual health education programs.

### Limitations

The methodology is subject to the constraints of purposive sampling, as well as social desirability bias, low male participation (husbands/dads), and small sample size. The first author (McGuire) lived and worked in the region for over a year, building close and trusted relationships within the community and developing a deep understanding of the context prior to this study. However, McGuire’s positionality in the study setting may still have had affected participant responses and prompted some social desirability bias [32]. To neutralize this, moderator neutrality was established from the beginning, conforming responses were further probed, and group processing techniques were used to build context and arrive at participants real motivations, barriers, and anxieties [33, 34]. The results in this paper reflect the views of 24 individuals in Amazonas, Peru, and these findings are not representative of all adolescents in rural areas of Peru. With that being said, the results provide insight into the SRH knowledge and attitudes of an understudied population. Given the relatively high number of adolescents living in rural areas in Peru, and other Andean and Amazonian regions, the findings from this paper could be used to inform future projects around adolescent SRH knowledge.

Future studies should recruit men and women from older age groups. Although they may be subject to recall bias, they may feel more comfortable discussing sexuality and less prone to social desirability bias. Future studies should also include health care providers as well as local teachers and use quantitative measures to further triangulate the data. Further investigation is required concerning actual health policy in the region. There are several ongoing programs with internal evaluations, which should undergo more rigorous analytic methodology. Finally, future research led by local adolescents (participatory or co-production research designs) could be used as a way of supporting this population in exploring what matters to them and creating a space which helps mitigate social desirability bias [3].

## Conclusion

Knowledge about SRH topics in these highland communities was limited. The findings from our study give much needed insight in a region with no published or available information about SRH knowledge and practices and may be relevant to other rural contexts in Peru and Latin America. The outcomes established in the study point towards a need for more SRH education, as well as identify existing trusted sources for information. This knowledge can be utilised when planning future public heath interventions for SRH. Due to some of the sensitive subjects that were touched on, more qualitative and ethnographic research which focuses on the cultural side of sexuality and sexual relations in the area would be beneficial to further explore and understand SRH in this context.

## Data Availability

The data represented in this study is not public due to participant privacy, but is available through the corresponding author upon reasonable request.

## Abbreviations

SRH: Sexual and reproductive health
FGD: Focus group discussions
PPI: Personal private interview
STI: Sexually transmitted infection
MoE: Ministry of Education

## Footnotes

1 This data was collected prior to the 2015–2016 Zika virus (ZIKV) epidemic in Latin America. While the Amazonas region had no confirmed cases of ZIKV, a health emergency was temporarily declared. Government funding was supplied for health promotion and awareness campaigns, with the bulk of the activities taking place in the Loreto region [19, 20].

2 In Amazonas, 52.3% of women are not married, but are *conviviendo, or* cohabitating [9]. As stated in the FGDs, marriage is expensive and there is little benefit for either partner.
